# Venom from *Bothrops lanceolatus*, a Snake Species Native to Martinique, Potently Activates the Complement System

**DOI:** 10.1155/2018/3462136

**Published:** 2018-07-15

**Authors:** Marie Delafontaine, Isadora Maria Villas-Boas, Giselle Pidde, Carmen W. van den Berg, Laurence Mathieu, Joël Blomet, Denise V. Tambourgi

**Affiliations:** ^1^Immunochemistry Laboratory, Butantan Institute, Av. Prof. Vital Brazil 1500, 05503-900 São Paulo, SP, Brazil; ^2^Prevor Laboratory, Moulin de Verville, 95760 Valmondois, France; ^3^Centre for Medical Education, Cardiff University, School of Medicine, Cardiff CF144XN, UK

## Abstract

*Bothrops lanceolatus* snake venom causes systemic thrombotic syndrome but also local inflammation involving extensive oedema, pain, and haemorrhage. Systemic thrombotic syndrome may lead to fatal pulmonary embolism and myocardial and cerebral infarction. Here, we investigated the ability of *B. lanceolatus* venom to activate the Complement system (C) in order to improve the understanding of venom-induced local inflammation. Data presented show that *B. lanceolatus* venom is able to activate all C-pathways. In human serum, the venom strongly induced the generation of anaphylatoxins, such as C5a and C4a, and the Terminal Complement complex. The venom also induced cleavage of purified human components C3, C4, and C5, with the production of biologically active C5a. Furthermore, the venom enzymatically inactivated the soluble C-regulator and the C1-inhibitor (C1-INH), and significantly increased the expression of bound C-regulators, such as MCP and CD59, on the endothelial cell membrane. Our observations that *B. lanceolatus* venom activates the three Complement activation pathways, resulting in anaphylatoxins generation, may suggest that this could play an important role in local inflammatory reaction and systemic thrombosis caused by the venom. Inactivation of C1-INH, which is also an important inhibitor of several coagulation proteins, may also contribute to inflammation and thrombosis. Thus, further *in vivo* studies may support the idea that therapeutic management of systemic *B. lanceolatus* envenomation could include the use of Complement inhibitors as adjunct therapy.

## 1. Introduction

Snakes from the genus *Bothrops* are responsible for the majority of venomous ophidian accidents in South and Central America [1]. They induce a complex pathophysiology, referred to as bothropic syndrome. After envenomation, haemorrhage, pain, and oedema appear quickly at the site of the bite, whereas coagulation disturbances, haemorrhage, and renal failure are commonly observed systemic symptoms [2]. Dermonecrosis, myonecrosis, and local infection can cause disabling sequels [3, 4].

*Bothrops lanceolatus*, commonly named Martinique lancehead (“Fer-de-lance”), is a native species confined to the Caribbean island of Martinique. Systemic clinical symptoms of *B. lanceolatus* envenomation differ from envenomation by other *Bothrops* species as it is characterized by a predominant prothrombotic profile and is rarely haemorrhagic [2]. In approximately 30–40% of the cases, multiple arterial thrombi occur, which is unique to *B. lanceolatus* and *B. carribeus*, a *Bothrops* species from the neighbouring island of Saint Lucia. This can lead to death due to myocardial and cerebral infarction or pulmonary oedema [5]. Local effects are comparable to the bothropic syndrome involving prominent oedema, pain, and haemorrhage from the fang marks. In case of envenomation, only rapid treatment with the monospecific commercial antivenom, raised against *B. lanceolatus* venom (Bothrofav®, Sanofi Pasteur, France), can prevent the development of systemic thrombosis, which if untreated can result in death [5, 6]. But even this antivenom is not always effective and supplementary therapies may be beneficial to the patient [6]. Thus, a better understanding of how this venom causes pathology is required.

*Bothrops* snake venoms are complex mixtures of bioactive organic and inorganic components, such as proteins, peptides, carbohydrates, lipids, and mineral salts. These venoms display a wide range of interspecies variations both in composition and biological activities [7–12]. Metallo- and serine proteases are among the most abundant enzymes found in *Bothrops* venoms. They play a central part in the local and systemic development of the pathophysiology of envenomation, respectively, by inducing haemorrhage, myonecrosis, inflammation, cutaneous lesions, and haemostasis disturbances [12–15].

The Complement (C) system, a complex group of more than 50 blood-circulating and cell-surface-expressed and intracellular proteins, is an important effector mechanism of innate and adaptive immunity [16]. Amplification of the inflammatory response, phagocytosis, lysis of pathogenic agents, and recognition of altered self are a few of the biological processes in which the Complement system is involved [17–20]. Once activated, a chain reaction of proteolysis and assembly of protein complexes evolves, which is finely regulated by soluble and membrane-bound regulators [21]. Complement activation can be initiated through any of its three activation pathways: classical (CP), alternative (AP), or lectin (LP), all converging towards the formation of C3-convertases and the cleavage of C3 component into C3b and the anaphylatoxin C3a. C3b is involved in the formation of the C5-convertase, which in turns cleaves C5 into C5b and the anaphylatoxin C5a. C5b interacts with C6, C7, C8, and several C9 proteins to form the membrane attack complex (C5b-9 or MAC), which generates a lytic pore in the target membrane. The anaphylatoxins C3a, C4a, and C5a constitute potent proinflammatory mediators, via the interaction with specific receptors such as C3aR and C5aR1 [22]. The activation of the Complement cascade is regulated by membrane proteins including Complement-receptor 1 (CR1), membrane cofactor protein (MCP/CD46), decay-accelerating factor (DAF/CD55), and CD59. Factor I, Factor H, Factor-H related proteins, C4-binding protein (C4BP), and C1-inhibitor (C1-INH) are important soluble regulators of Complement [23–26].

Inappropriate activation of the Complement cascade can be harmful to the host and can lead to inflammation and thrombosis [27]. Numerous animal venoms interact with the human Complement system, for example, by initiating the C-cascade activation, as shown for venoms of 19 different *Bothrops* species from South and Central America [28, 29]. In these studies, we showed that *Bothrops* venoms triggered the C-cascade by one or several activation pathways, generating high quantities of anaphylatoxins by directly cleaving C3 and C5 or by inactivating the regulator C1-INH. These events involved both metalloproteases and serine proteases. No modifications of the membrane regulators, DAF, CR1, and CD59, were observed in case of *Bothrops* venoms exposure [28, 30–32]. The activation of the Complement system by *Bothrops* venoms may constitute an important event in human bothropic envenomation pathophysiology.

*B. lanceolatus* venom is antigenically similar to other *Bothrops* venoms but differs from other *Bothrops* venoms in that it does not induce coagulation of human plasma, has very low hyaluronidase activity, and their proteases have different substrate specificities [10, 33]. The *B. lanceolatus* venom contains glycosylated proteins that could potentially trigger the lectin Complement pathway [10, 34]. Considering that the anti-*B. lanceolatus* antivenom Bothrofav is not always 100% percent effective at preventing local and systemic events [6], supplementary therapies may be beneficial to the patient. Thus, a better understanding of how this venom causes inflammation and thrombosis is required. Considering the important role Complement can play in inflammation and thrombosis, we investigated the potential of *B. lanceolatus* to activate the Complement cascade *in vitro*, and interact with its proteins, regulators, and receptors. Here, we show that *B. lanceolatus* venom activates Complement cascade, affecting major C-components and soluble/membrane-bound inhibitors, and inducing the production of proinflammatory mediators.

## 2. Material and Methods

### 2.1. Chemicals, Reagents, and Buffers

Tween-20, Dextran 70, ethylene diamine tetraacetic acid (EDTA), ethylene glycol-bis-(beta-aminoethylether)-N,N,N′,N′-tetraacetic acid (EGTA), barbituric acid, sodium barbital, *ortho*-phenylenediamine (OPD), phenylmethanesulfonyl fluoride (PMSF), 1,10-phenantroline, mannan, and bovine serum albumin (BSA) were purchased from Sigma-Aldrich (St. Louis, MO, USA). Purified human Complement components C3, C4, C5, and C1-INH, as well as goat anti-human C4 antibody were purchased from Quidel Corporation (San Diego, CA, USA). Fluo-4 AM was from Life Technologies (Waltham, MA, USA). The following murine antibodies against human CD46 (clone E4.3), CD55 (clone IA10), and CD59 (clone p282-H19) were purchased from BD Pharmingen (San Diego, CA, USA). Polyclonal goat anti-mouse IgG-PE, polyclonal rabbit anti-goat IgG labelled with alkaline phosphatase, and goat anti-human C1-INH antibodies were obtained from Sigma-Aldrich (MO, USA). Foetal bovine serum (FBS) was from Cultilab (São Paulo, Brazil). Dulbecco's modified Eagle's medium (DMEM), penicillin, and streptomycin were from Gibco/Thermo Fisher Scientific (Gaithersburg, MD, USA). Buffers include saline solution (150 mM NaCl), PBS (8.1 Mm Na_2_HPO_4_, 1.5 mM KH_2_PO_4_, 137 mM NaCl, and 2.7 mM KCl, pH 7.2), Alsever's solution (114 mM citrate, 27 mM glucose, 72 mM, and NaCl, pH 6.1), Veronal-Buffered Saline (VBS^2+^; 2.8 mM barbituric acid, 145.5 mM NaCl, and 0.3 mM CaCl_2_, pH 7.2), AP buffer (5 mM sodium barbital, 7 mM MgCl_2_, 10 mM EGTA, and 150 mM NaCl, pH 7.4), BVB^2+^ buffer (VBS^2+^, 0.5 mM MgCl_2_, 2 mM CaCl_2_, 0.05% Tween-20, and 0.1% BSA, pH 7.5), BSS buffer (137 mM NaCl, 2.68 mM KCl, 78.3 mM Na_2_HPO_4_, and 1.47 mM KH_2_PO_4_), Krebs buffer (120 mM NaCl, 25 mM HEPES, 4.8 mM KCl, 1.2 mM KH_2_PO_4_, and 1.2 mM MgSO_4_), and FACS buffer (1% BSA, 0.01% NaN_3_ in PBS).

### 2.2. Venom

Venom from *Bothrops lanceolatus* (*B. lanceolatus*) was obtained from Latoxan (Aix-en-Provence, France). Stock solution was prepared in sterile saline solution at 5 mg/mL and stored at −80°C.

### 2.3. Normal Human Serum, Erythrocytes, and Ethics Statements

Human blood was obtained by signed consent from healthy donors. Blood was collected by venepuncture without anticoagulant and refrigerated for 4 h. After clot formation, blood was centrifuged at 400 ×g for 15 min at 4°C, serum was collected, aliquoted, and stored at −80°C. This study was approved by the Human Research Ethics Committee (HREC) from the Institute of Biomedical Sciences at the University of São Paulo, São Paulo, Brazil (protocol approval number 274.313). Sheep and rabbit erythrocytes were collected in Alsever's. All the procedures involving animals were in accordance with the ethical principles in animal research adopted by the Brazilian Society of Animal Science and the National Brazilian Legislation number 11.794/08. Protocols were approved by the Institutional Animal Care and Use Committee (protocol approval number 1111/13).

### 2.4. Treatment of Normal Human Serum with *B. lanceolatus* Venom

Normal human serum (NHS) was incubated with the same volume of *B. lanceolatus* venom in the appropriate buffer (i.e., for CP: VBS^2+^; AP: APB; and LP: BVB^2+^), at 37°C for 30 min, to assess effects on the Complement system. Samples were tested for the remaining Complement activity or detection of the activation by-product. The role of metalloproteases in the venom was investigated by coincubation with 1,10-phenantroline (10 mM).

### 2.5. Haemolytic Complement Assays

Haemolytic activity was assessed using sensitized sheep erythrocytes (E_S_) for the CP [35], or rabbit erythrocytes (E_R_) for the AP [28]. CH_50_ and AP_50_ were calculated for each venom concentration. Venom concentrations able to inhibit 50% (IC_50_) of the activity of each pathway and their respective 95% confidence intervals (CI95%) were determined from the curves of CH_50_ and AP_50_ plotted as a function of the logarithm of venom concentration by nonlinear regression using GraphPad Prism 6.0 software (GraphPad, La Jolla, CA, USA) [32, 35].

### 2.6. Action of *B. lanceolatus* Venom on Lectin Pathway

LP activity was assessed using mannan-coated microtiter plates and measurement of C4 deposition as previously described [28]. LP_50_ was calculated for each venom concentration. Venom concentrations able to inhibit 50% (IC_50_) of the activity of LP and its 95% confidence interval (CI95%) were determined from the values of LP_50_ for each venom concentration (see Section 2.5) by nonlinear regression using GraphPad Prism 6.0 software.

### 2.7. Detection of Anaphylatoxins and Terminal Complement Complex (TCC) in Venom-Treated Samples

C3a/C3a-desArg, C4a/C4a-desArg, and C5a/C5a-desArg were measured using the Human Anaphylatoxin Cytometric Bead Array (CBA) (BD Biosciences Pharmingen, San Diego, CA, USA), following the manufacturer's instructions. Cytometric analysis was performed using a FACSCanto-II (Becton Dickinson, San Diego, CA, USA), and the data were analysed with the Flow Cytometric Analysis Program (FCAP) Array 3.0 (Becton Dickinson, San Diego, CA, USA). Anaphylatoxin concentrations (*μ*g/mL) were determined by linear regression from the standard curve. TCC (in its soluble form SC5b-9) was determined using the MicroVue™ SC5b-9 Plus EIA Kit (Quidel Corporation, San Diego, CA, USA), according to the manufacturer's instructions. TCC concentration (*μ*g/mL) in the samples was calculated from a linear regression of the standard curve.

### 2.8. Cleavage of Purified Complement Components by *B. lanceolatus* Venom

To evaluate the direct cleavage of the Complement components by the *B. lanceolatus* venom, purified human Complement proteins, C3, C4, C5, and C1-INH (2 *μ*g from 0.2 mg/mL samples), were incubated with the same quantity of venom for 30 min at 37°C. C1-INH was incubated with venom in the presence or absence of protease inhibitors, PMSF (20 mM), EDTA (20 mM), or 1,10-phenantroline (20 mM). Samples were then submitted to SDS-PAGE (10% separating gel) under reducing conditions and silver-stained to detect cleavage [36, 37]. In addition, cleavage of C1-INH was also revealed by Western blotting, using goat anti-human C1-INH antibodies (1 : 2000) and rabbit anti-goat IgG labelled with alkaline phosphatase (1 : 7500). The reaction was developed by adding NBT/BCIP, following the instructions of the manufacturer.

### 2.9. Inhibitory Activity of C1-INH following Incubation with *B. lanceolatus* Venom

C1s-binding activity of C1-INH was assessed using the MicroVue™ C1-Inhibitor Plus EIA (Quidel Corporation, CA, USA), according to the manufacturer's procedure. Samples of purified human C1-INH were exposed to *B. lanceolatus* venom. As control, saline-treated C1-INH samples were used. Following the assay procedure, samples (diluted 1 : 25) were incubated with biotinylated C1s (venom concentration of 8 *μ*g/mL), and were incubated on avidine-coated microtiter plates. Horseradish peroxidase- (HRP-) conjugated goat anti-human C1-INH was added to each test well and the reaction developed enabling the detection of C1-INH-C1s complexes deposited on the plates. After subtraction of the blank, the residual C1-INH-C1s-complexing activity of each sample was expressed as a percentage of the control sample activity.

### 2.10. Intracellular Calcium Flux Measurements

Functional C5a generation was assessed by measuring cytoplasmic calcium release from intracellular stores in leukocytes, of which neutrophils are the main population and the main responders, using the calcium indicator Fluo-4 AM. Leukocytes were separated from platelets and red blood cells by dextran sedimentation from heparinised human blood using standard procedures. Cells were resuspended in Dulbecco's modified Eagle's medium (DMEM), 10% heat-inactivated foetal bovine serum (FBS), and loaded with the fluorescent calcium indicator Fluo-4 AM (1 *μ*M) at 2 *μ*g/10^7^ cells/mL, at room temperature for 30 min. Cells were washed with BSS and resuspended in Krebs buffer containing 0.1% BSA. Cells (10^6^/well in 200 *μ*L Krebs buffer) were stimulated with venom-treated C5 (10 *μ*L/well; final concentration of 9.5 *μ*g/mL), in a 96-well plate and the fluorescence variations were measured every 3 sec (*λ*_ex_ = 485 nm and *λ*_em_ = 510 nm), using the fluorimeter FLUOstar Omega (BMG Labtech, Offenburg, Germany). As positive control, cells were exposed to purified C5a (10 *μ*L/well; final concentration of 11.4 ng/mL) and, as negative controls, cells were exposed to C5 or venom incubated with saline solution. For each well, the values of fluorescence were normalized by the initial fluorescence F_0_, the average fluorescence during 30 sec before stimulation, and then plotted against time.

### 2.11. Cell Culture and Flow Cytometry

The endothelial cell line EA.hy926 was cultured in DMEM containing 10% (*v*/*v*) heat-inactivated FBS, and the antibiotics, penicillin, and streptomycin (100 IU/mL) at 37°C in humidified air with 5% CO_2_. Cells were harvested using trypsin/EDTA and incubated at 106 cells/mL with 100 *μ*g/mL of *B. lanceolatus* venom, for 2 h at 37°C under constant agitation. Cells were centrifuged at 400 ×g and resuspended in FACS buffer. Cells (5 × 10^4^ cells/well) were incubated for 1 h at 4°C with monoclonal antibodies anti-human MCP, DAF, or CD59 (1 *μ*g/mL), followed by incubation with goat anti-mouse IgG-PE (1 : 100), for 1 h at 4°C. Cells were analysed by flow cytometry (FACSCanto-II, Becton Dickinson, San Diego, CA, USA) using the software FACSDiva (Becton Dickinson, San Diego, CA, USA).

### 2.12. Statistics

Numerical data were expressed as mean ± SD. Data were analysed statistically by Student's *t*-test, or by one way ANOVA and Bonferroni's multiple comparison test, using the software GraphPad Prism 6 (GraphPad Software, Inc., La Jolla, CA, USA). A *P* value < 0.05 was considered significant.

## 3. Results

### 3.1. *B. lanceolatus* Venom Affects All Three Complement Activation Pathways

**Figure 1:**
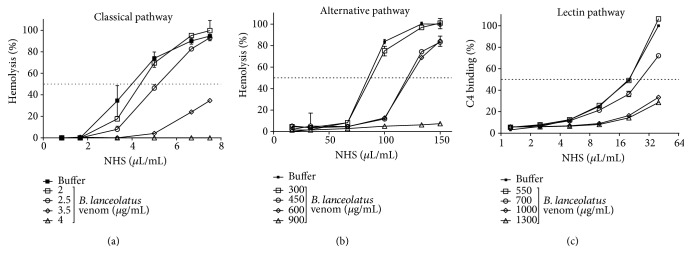
Activation of the three Complement pathways by *B. lanceolatus* venom. NHS (dilution 1 : 80 (a), 1 : 4 (b), and 1 : 25 (c)) was incubated with several concentrations of *B. lanceolatus* venom at 37°C for 1 h (CP) or 30 min (AP and LP), and diluted to test their residual Complement activity. NHS incubated only with buffer was used as control. (a) Sensitized sheep erythrocytes were used to assess the CP while (b) rabbit erythrocytes (E_R_) were used for the AP. (c) For the LP, residual binding of C4 on a mannan-coated plate was used. The results represent mean ± SD of duplicates of a representative assay. Experiments were performed three times. The CH_50_, AP_50_, and LP_50_ were calculated by nonlinear regression from these curves. The IC_50_ of the venom for each pathway and respective CI95% were calculated. The venom affected the classical pathway with an IC_50_ of 156.6 *μ*g/mL of NHS (CI95%: 147.3–166.6 *μ*g/mL of NHS), the alternative pathway with an IC_50_ of 294.5 *μ*g/mL of NHS (CI95%: 273.2–317.4 *μ*g/mL of NHS), and the LP pathway with an IC_50_ of 396.3 *μ*g/mL of NHS (CI95%: 375.7–418.0 *μ*g/mL of NHS).

Human serum (NHS) was incubated with *B. lanceolatus* venom and residual haemolytic activity was assessed. As shown in Figures 1(a) and 1(b), the venom dose-dependently reduced the lytic activity of CP and AP of NHS. The venom affected the classical pathway with an IC_50_ of 156.6 *μ*g/mL (CI95%: 147.3–166.6 *μ*g/mL) and the alternative pathway with an IC_50_ of 294.5 *μ*g/mL (CI95%: 273.2–317.4 *μ*g/mL) (Figures 1(a) and 1(b)). The venom also affected the C4 deposition in the LP assay, resulting in an IC_50_ of 396.3 *μ*g/mL (CI95%: 375.7–418.0 *μ*g/mL) (Figure 1(c)).

### 3.2. *B. lanceolatus* Venom Generates C4a and C5a and TCC in NHS but Reduces C3a

**Figure 2:**
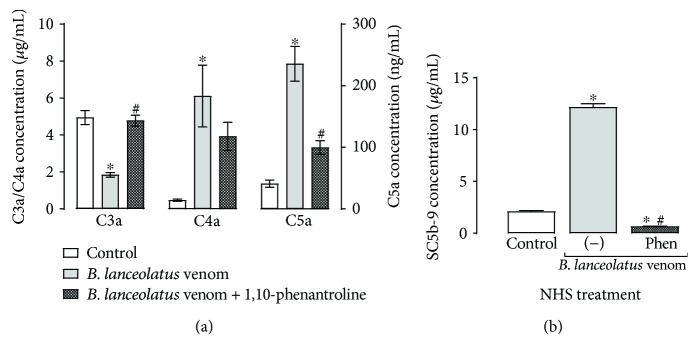
Production of Complement activation products in NHS incubated with *B. lanceolatus* venom. NHS (diluted 1 : 2 in saline solution) was incubated with *B. lanceolatus* venom (0.5 mg/mL) for 30 min at 37°C, in the presence or absence of 1,10-phenantroline (10 mM). As control, NHS was incubated with saline solution. (a) Samples were diluted 1 : 5000 and the concentrations of the anaphylatoxins C3a/C3a-desArg, C4a/C4a-desArg, and C5a/C5a-desArg were determined using a cytometric bead array. (b) Samples were diluted 1 : 150, and the concentration of the SC5b-9 complex was measured by “MicroVue SC5b-9 Plus EIA Kit”. The data represent two experiments, realized in duplicate. ^∗^*p* < 0.05 compared to the saline-treated NHS controls. ^#^*p* < 0.05 compared to venom-treated samples.

To establish whether the observed reduction C-activity resulted from inhibition or activation (resulting in consumption) of the Complement cascade by the venom, the presence of anaphylatoxins and terminal Complement complex (TCC) in venom-treated NHS samples was investigated.  Figure 2 reveals that *B. lanceolatus* venom induces a significant production of C4a, C5a, and TCC, thus confirming direct activation of the Complement system by the venom. Surprisingly, the venom caused a significant decrease of C3a in the serum, when compared with the control. These activities were inhibited with the addition of a metalloprotease inhibitor, 1,10-phenantroline, suggesting the participation of *B. lanceolatus* venom metalloproteases in Complement activation.

### 3.3. *B. lanceolatus* Venom Cleaves Purified C3, C4, and C5 and Generates Active C5a

**Figure 3:**
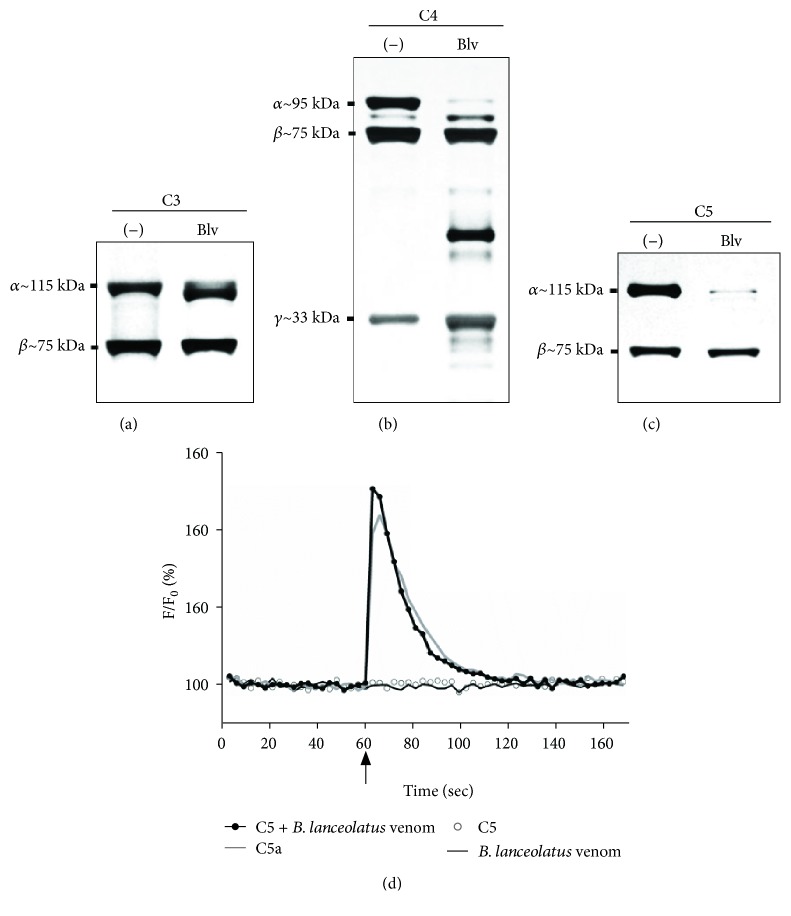
Cleavage of purified C3, C4, and C5 by *B. lanceolatus* venom. Samples (2 *μ*g) of purified Complement components C3 (a), C4 (b), and C5 (c) were incubated in PBS with *B. lanceolatus* venom (2 *μ*g) for 30 min at 37°C. The cleavage was visualized by electrophoretic separation (10% acrylamide SDS-PAGE) under reducing conditions, followed by silver staining. (d) The functional activity of the venom-treated C5 fragments was assessed via measuring calcium influx in Fluo-4-AM-labelled leukocytes (5 × 10^6^ cells/mL). Purified C5a (2.4 ng/mL) and samples of C5 or venom incubated with saline were used as positive and negative controls, respectively. Cells were allowed to equilibrate for 60 sec before stimulation (arrow).

**Figure 4:**
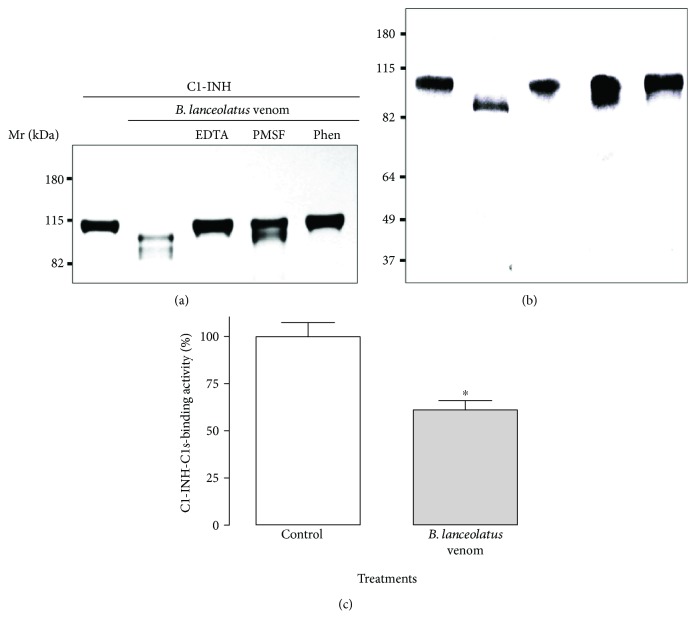
Cleavage of purified Complement regulator C1-INH by *B. lanceolatus* venom and its residual C1s-complexing activity. C1-INH (2 *μ*g) samples were incubated with *B. lanceolatus* venom (2 *μ*g) for 30 min at 37°C and the cleavage was visualized by SDS-PAGE electrophoresis, followed by silver staining (a), or by Western blot using anti-C1-INH antibodies (b). The residual C1s-complexing activity of the C1-INH fragments was assessed by functional ELISA (c). The results represent the mean of two independent experiments, carried out in triplicates. Data are represented as mean ± SD. ^∗^*p* < 0.05 between two samples.

Potential direct proteolytic action of the venom on the Complement components were investigated using purified C3, C4, and C5 and incubations with *B. lanceolatus* venom, in the presence or absence of proteases inhibitors. SDS-PAGE (Figures 3 and 4) shows that the venom reduced the intensity of staining of the *α*-chains of C3, C4, and C5 while generating fragments of slightly lower molecular mass, a pattern similar to what is usually observed when these molecules are activated via any of the Complement cascade. *β*- and *γ*-chains were not significantly affected.

To further investigate if the C5a fragment produced by the direct action of venom was functional, we measured its ability to activate leukocytes, by means of monitoring the calcium influx induced by C5a in these cells. Neutrophils constitute the major cell type in the leukocyte preparations and are the main and strongest responders to C5a in leukocyte preparations. Leukocytes, preloaded with the calcium sensor Fluo-4 AM, were exposed to venom-treated C5 samples.  Figure 3(d) demonstrates that venom-treated C5, similar to purified C5a, is able to induce a calcium influx in leukocytes, demonstrating that functionally active C5a was generated. Venom on its own or purified C5 on its own, did not induce a change in intracellular calcium.

### 3.4. *B. lanceolatus* Venom Cleaves and Partially Inactivates C1-INH

We previously showed that venoms from a range of *Bothrops* snakes caused the cleavage of C1-INH, the soluble regulator of the classical and lectin pathways. We show here that *B. lanceolatus* venom also has that ability (Figure 4). Both silver-staining (Figure 4(a)) and Western blotting (Figure 4(b)) showed that the single-chain C1-INH (reported to have a Mr of 105 kDa [38]) was reduced in Mr by the venom action (with a reduction in size to approximately 98 kDa). This activity was totally inhibited by the metalloprotease inhibitors, EDTA and 1,10-phenantroline, whereas the inhibition displayed by the serine protease inhibitor, PMSF, was partial (Figures 4(a) and 4(b)). Using a functional ELISA assay testing the binding of C1s, we observed that the venom-generated C1-INH fragments had a reduced binding activity to C1s (Figure 4(c)), as compared to the nonvenom-treated purified component, indicating that it was inactivated by the venom.

### 3.5. *B. lanceolatus* Venom Does Not Reduce Expression of Membrane-Bound C-Regulators

**Figure 5:**
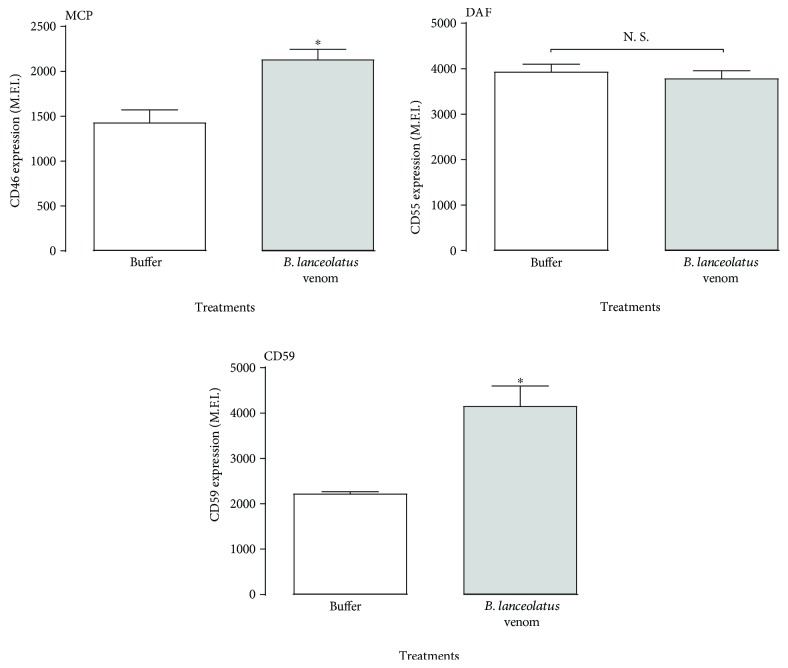
Effect of *B. lanceolatus* venom exposure on C-regulator expression on endothelial cells. EA.hy926 cells (10^6^ cells/mL) were incubated with *B. lanceolatus* venom (100 *μ*g/mL) for 2 h. After incubation with DAF-, MCP-, and CD59-specific antibodies, the fluorescence was assessed by flow cytometry. The results are representative of two experiments, carried out in triplicates. Data are represented as mean ± SD. ^∗^*p* < 0.05 compared to the buffer-treated controls. ^#^*p* < 0.05 between two samples.

The Complement system also includes regulatory proteins present on the cell membranes, and reduction in expression on endothelial cells would make cells more susceptible to C-induced activation. Using the endothelial cell line EA.hy926, we investigated the effects of *B. lanceolatus* venom on the expression of membrane-bound C-regulators DAF, MCP, and CD59. The venom significantly increased the expression of MCP and CD59 and did not affect DAF expression (Figure 5).

## 4. Discussion

*B. lanceolatus*, commonly called the Martinique lancehead and Martinican pit viper, is the only endemic snake on Martinique [1, 2]. Clinical presentations of envenomations by *B. lanceolatus* are characterized by systemic thrombotic syndrome and important local inflammation, involving oedema and pain, but limited haemorrhage.

The Complement system is an important contributor to and amplificator of inflammation if activated in excess or inappropriately controlled. Anaphylatoxins generated as a consequence of C activation are major contributors to the inflammation. The Complement system can also contribute to thrombosis by activating endothelial cells and contributing to platelet activation, and both anaphylatoxins and the TCC are contributors [27]. In this study, we analysed the action of *B. lanceolatus* venom on the Complement system activation pathways, components, regulators, and receptors. We show here that the venom can activate all three Complement pathways (Figure 1). Activation of the AP may be simply a consequence of activation of the LP and CP, which often also results in activation of the AP, which then acts as an amplification loop. Recently, we have demonstrated the presence of glycosylated proteins in this venom [10], which potentially are involved the lectin pathway activation; however, this remains to be investigated.

Complement activation by *B. lanceolatus* venom was also demonstrated by the detection of C-activation products, such as the anaphylatoxins C4a and C5a and TCC (Figure 2). However, a decrease rather than an increase in the concentration of C3a was detected in venom-treated NHS. We have previously shown that out of venoms from 19 different *Bothrops* species, 18 induced the generation of C3a in serum, but similar to *B. lanceolatus*, the *B. brazili* venom also induced a reduction in C3a [28]. These results may be due to the presence of venom peptidases able to further hydrolyse C3a. C3a is a potent proinflammatory signal for several cell types, such as macrophages/monocytes, peripheral nonadherent PBMCs, and mast cells [39]. In contrast to C5a, C3a does not directly activate neutrophils and can prevent their mobilization from bone marrow to the blood stream, which constitutes an anti-inflammatory action in the early phase of inflammation [39–41]. Thus, the reduction of C3a and the increase of C5a in *B. lanceolatus* venom may contribute to its potent proinflammatory action. However, further kinetics studies analysing C3a and C5a generation and degradation, are necessary to define the possible consequences of these results in the envenomation process.

Analysis of the contributions of metalloproteases, common components of *Bothrops* venoms, showed that the metalloprotease inhibitor 1,10-phenanthroline partially or completely prevented the effects of the venom on the generation of anaphylatoxins and TCC in whole serum (Figure 2), suggesting an important role for metalloproteases in the activation of the Complement system. The role of serine proteases in this process cannot be investigated as the enzymes in the Complement system themselves are serine proteases.

Considering that *Bothrops* venoms contain a large amount of proteases, we analysed the ability of *B. lanceolatus* venom to directly cleave the sources of anaphylatoxins, such as C3, C4, and C5. Data presented here show that venom components can hydrolyse the *α*-chain of the three Complement proteins (Figure 3). These fragments could participate or be the origin of the potent activation of the three pathways by the venom; however, their functional activity remains to be tested. Purification of venom components would be required to investigate the identity of the components responsible for Complement activation and enzymatic cleavage of purified components.

We also show here that the C5a fragment, generated through the degradation of purified C5 by *B. lanceolatus* venom, is functionally active, demonstrating that venom proteases directly cleave C5 into functional C5a (Figure 3(d)). The cleavage of C5 after envenomation, due to the generation of a C5-convertase, as a consequence of the activation of the three Complement pathways, is likely an important mechanism in the generation of C5a (Figures 1 and 2). In addition, direct activation of C5a is a possibility, and thrombin-like enzymes have already been reported in *Bothrops* sp. venoms [42–44] and may be responsible for the direct generation of C5a, since human thrombin can cleave C5 into functional C5a. As activated human coagulation proteins (e.g., thrombin, plasmin, FX, and FXIa) are known to activate C5 [42, 45], further C5a could also be generated. Considering that both C5a [46–48] and TCC [49–51] can display several prothrombotic effects, how these two C-activation products contribute to the prothrombotic pathology observed in *B. lanceolatus* envenomation requires further investigation.

The C1-inhibitor, C1-INH, is the only known serine protease plasma inhibitor. It regulates not only the Complement cascade (inhibitor of C1r, C1s, and MASPs) but is also an important regulator of the coagulation cascade activation and inhibits several fibrinolytic proteins (kallikrein, FXIIa, FXIa, and plasmin) [52]. It inhibits the Complement serine protease C1s by acting as a pseudosubstrate: C1-INH is cleaved at the peptide-bound R444-T445 and forms a stable complex with C1s via its residues Q452, Q453, and F455 [53]. A deficiency of C1-INH can result in autoactivation of these pathways [54]. Our results reveal that *B. lanceolatus* venom also cleaves purified C1-INH, mainly by metalloprotease action, which may explain the activation of the C-cascades. In conditions of complete C1-INH conversion by *B. lanceolatus* venom, the C1s-inhibitory potential loss was about 40%, similar to the value observed with *B. pirajai* metalloprotease, C-SVMP, under comparable conditions [32]. This loss of function could result from C1s-binding site impairment by the venom. It could be related to the activation of classical and lectin pathways by *B. lanceolatus* venom. Furthermore, C1-INH is also an important regulator of several proteins involved in the coagulation cascade and dysfunction or deficiency of C1-INH can lead to excess generation of bradykinin, a potent vasodilator and important contributor to inflammation, and is associated with hereditary angioedema (HAE) [52]. This may be the major mechanism of how *B. lanceolatus* venom induces inflammation.

As Complement activation on endothelial cells can lead to a more prothrombotic phenotype of these cells [55], endothelial cells express Complement inhibitors on their membrane, such as membrane cofactor protein (MCP/CD46), decay-accelerating factor (DAF/CD55), and CD59 [56]. A deficiency of MCP has been associated with atypical human uraemic syndrome (aHUS) which is characterized by thrombotic microangiopathies [57], while a deficiency of DAF/CD59 is associated with paroxysmal nocturnal haemoglobinuria (PNH), which also is associated with thrombosis [58]. Furthermore, we previously reported the cleavage of MCP induced by *Loxosceles* spider venom [59] and this might contribute to the systemic thrombotic events associated with envenomation. In our study, the *B. lanceolatus* venom did not reduce expression of any of these regulators but, significantly, increased the detection of MCP and CD59 (Figure 5). Thus, increased thrombosis after *B. lanceolatus* envenomation is unlikely due to inefficient C regulation on the endothelial cells, but endothelial activation in response to excess C5a or TCC generation, as a consequence of the effect of the venom on the Complement system, may contribute. *Bothrops* species venoms activate the Complement cascade by several pathways and complex mechanisms [28]. In the case of *B. asper* and *B. pirajai* venoms, class I metalloproteases are involved [30, 32]. Since structural similarities exist between *Bothrops* toxins, *B. lanceolatus* PI-SVMPs may be involved in *B. lanceolatus* Complement activation, but this remains to be investigated.

In conclusion, here we show that, like its continental counterparts, *B. lanceolatus* venom is a potent trigger of the Complement cascade *in vitro*. Thus, further *in vivo* studies may support the idea that therapeutic management of systemic *B. lanceolatus* envenomation could include the use of Complement inhibitors as adjunct therapy.
